# Coronary Calcium Scoring as Prediction of Coronary Artery Diseases with Low-Dose Dual-Source CT

**DOI:** 10.3390/jcdd12110425

**Published:** 2025-10-27

**Authors:** Enrico Schwarz, Valentina Tambè, Silvia De Simoni, Roberto Moltrasi, Matteo Magazzeni, Elena Ciortan, Stefano Bentivegna, Anastasia Esseridou, Francesco Secchi

**Affiliations:** 1 Unit of Cardiology, Casa di Cura Igea, 20129 Milan, Italy; 2 Cardiovascular Imaging Unit, IRCCS Multimedica, 20099 Sesto San Giovanni, Italy; 3 Unit of Radiology, Casa di Cura Igea, 20129 Milan, Italy; 4 Department of Radiology, ASST Rhodense, 20024 Garbagnate Milanese, Italy; 5 Department of Biomedical Sciences for Health, Università degli Studi di Milano, 20122 Milan, Italy

**Keywords:** calcium scoring, coronary arteries disease, computed tomography, risk assessment, calcified plaques

## Abstract

The aim of this paper is to evaluate the correlation between the coronary calcium score (CCS) and coronary artery disease (CAD), patients underwent coronary CT angiography (CTA). Four hundred and five patients who underwent a coronary CT with CCS analysis were considered for this retrospective study. Coronary CTA was performed using a dual-source (256-slice) CT scanner (SOMATOM Definition Flash, Siemens Healthcare, Forchheim, Germany). Before injecting the contrast medium, non-contrasted cardiac CT was performed in a longitudinal scan field from the tracheal carina down to the diaphragm. The corresponding images for calcium scoring were reconstructed with a slice width of 1.5 mm and a slice interval of 1 mm, and the tube voltage was 120 kVp. The total calcium score was calculated using dedicated software. The calcium score based on the Agatston method was defined as the presence of a lesion with an area greater than 1 mm^2^ and peak intensity greater than 130 Hounsfield Units, which was automatically identified and marked with color by the software. From the radiological report, the degree of coronary stenosis was retrieved. A score of 1 corresponds to the absence of stenosis, a score of 2 to mild stenosis (<50%), and a score of 3 to moderate/severe stenosis (>50%). The total coronary gravity score (CGS) for each patient was calculated by summing the score of each coronary artery. The Spearman test was used for correlation. Out of the 405 patients, 217 were male. The mean and standard deviation age was 72 ± 11 years. The overall amount of calcium was an Agatston score of 393 ± 709. A positive correlation between CCS and CGS was found (r = 0.835 and *p* < 0.001). A ROC curve with AUC 0.917 (*p* ≤ 0.001) was obtained. The optimal cutoff point of the calcium score for discriminating CGS < 2 was 112, yielding sensitivity of 90% and specificity of 81%. This study confirms the important relationship between the coronary artery calcium score and the presence and extension of coronary artery disease.

## 1. Introduction

Coronary artery computed tomography angiography (CTA) has emerged as one of the most advanced and widely used non-invasive imaging techniques for the evaluation of coronary artery disease (CAD). With rapid and continuous advancements in computed tomography (CT) technology—particularly the development of dual-source CT scanners and high-pitch spiral acquisition—CTA now allows for high-resolution, three-dimensional imaging of coronary vessels. This detailed visualization enables the detection and characterization of coronary artery lesions, including luminal stenoses, non-calcified plaques, and vascular remodeling [1]. Such capabilities make coronary CTA a highly effective diagnostic alternative to invasive coronary angiography (ICA), which involves catheter-based contrast injection directly into the coronary arteries, carrying potential procedural risks such as vascular injury, bleeding, arrhythmias, and contrast-induced nephropathy, as well as requiring hospital admission and post-procedural monitoring. A crucial strength of coronary CTA lies in its negative predictive value, particularly in patients with low to intermediate pre-test probability of CAD. In such clinical scenarios, CTA can confidently exclude the presence of significant coronary artery stenosis, thereby avoiding the need for invasive diagnostic procedures [2]. This approach not only reduces patient exposure to procedural risk but also optimizes healthcare resource allocation and reduces costs, all while providing accurate and clinically useful diagnostic information. In many modern cardiology practices, CTA has become a first-line imaging modality in selected patient populations, thanks to its ability to detect both obstructive and non-obstructive coronary disease [3,4]. However, despite its diagnostic strengths, coronary CTA has notable limitations, especially in patients with extensive coronary artery calcification (CAC). High levels of calcification can significantly impair image interpretation, as calcified plaques create artifacts—commonly referred to as “blooming”—which obscure the lumen of the vessel and complicate the assessment of stenosis. In these cases, the presence of calcium can lead to overestimation of stenosis severity, potentially resulting in unnecessary invasive testing or overtreatment [5,6]. Therefore, while CTA is a highly valuable tool, its diagnostic accuracy can be compromised in individuals with advanced atherosclerosis marked by significant calcific burden. The phenomenon of coronary artery calcification is not only a technical challenge for CTA but also a biological hallmark of atherosclerotic cardiovascular disease. CAC is the result of calcium deposition in the intimal and medial layers of coronary vessels, typically occurring over years or decades of exposure to cardiovascular risk factors. These include hypertension, hyperlipidemia, diabetes mellitus, smoking, obesity, and chronic systemic inflammation. As such, the presence of CAC reflects the cumulative burden of atherosclerotic disease and serves as a surrogate marker for cardiovascular risk, even in asymptomatic individuals [7,8]. To quantify this burden, the coronary calcium score (CCS)—first introduced by Agatston and colleagues in the early 1990s—has become a standard method of assessing subclinical coronary atherosclerosis using non-contrast CT. The Agatston score is calculated based on both the area and the density of calcified plaques within the coronary arteries [9]. Each calcified lesion is scored and summed across the major coronary arteries to generate a total CCS. This score has been validated across multiple large cohorts as a powerful independent predictor of future cardiovascular events. Studies consistently show a graded relationship between increasing CCS values and the incidence of major adverse cardiac events (MACEs), including myocardial infarction, unstable angina, and cardiac death [9]. For instance, individuals with a CCS of 0 have a very low risk of future coronary events—often referred to as the “power of zero”—whereas those with scores exceeding 100, 400, or 1000 demonstrate progressively elevated risk. Notably, a calcium score above 400 is widely accepted as indicative of severe coronary calcification and correlates with the presence of advanced CAD [9]. In such cases, further diagnostic evaluation using CTA or invasive angiography is generally warranted to assess the degree of luminal obstruction and guide therapeutic decisions [9,10]. Despite the robust evidence supporting the prognostic value of CCS, it remains underutilized in routine cardiovascular risk stratification [10]. Most traditional risk scores, such as the Framingham Risk Score, SCORE, and ASCVD calculator, rely heavily on clinical variables like age, sex, blood pressure, lipid levels, and smoking status, without incorporating direct measures of atherosclerotic burden. This can lead to both overestimation and underestimation of true risk, particularly in individuals with subclinical disease. Incorporating CCS into these models has been shown to improve risk classification and help guide preventive therapies, such as the initiation of statins or antiplatelet agents. However, the integration of CCS into clinical guidelines remains incomplete, in part due to ongoing debates about the cost-effectiveness, optimal timing, and appropriate patient populations for calcium scoring. Moreover, the relationship between high CCS values and long-term outcomes in specific subgroups—such as women, ethnic minorities, or patients with diabetes or chronic kidney disease—is still being investigated, highlighting the need for more granular data and population-specific validation. The presence of coronary calcification remains a major obstacle to accurate stenosis assessment. When calcium deposition is extensive, the resulting artifacts can blur vessel borders and mimic high-grade stenoses, potentially leading to false-positive results. This limitation underscores the importance of interpreting CTA in conjunction with CCS and other clinical parameters. For patients with very high calcium scores, invasive coronary angiography may still be required for definitive diagnosis, particularly if symptoms persist or if non-invasive findings remain inconclusive. Given these challenges, researchers have explored alternative approaches to enhance CTA performance in heavily calcified arteries. These include advanced image post-processing techniques, dual-energy CT, and artificial intelligence-based algorithms that can better distinguish calcium from contrast-enhanced lumen. While promising, these innovations are still under development and not yet widely implemented in routine practice. Despite the technical limitations posed by calcification, the diagnostic role of CCS remains central. In fact, some studies suggest that combining CCS and CTA findings provides a more comprehensive picture of coronary health than either modality alone. For example, a patient with chest pain and a CCS of 0 is unlikely to have obstructive CAD, even if minor non-calcified plaques are present. Conversely, a high CCS should prompt clinicians to interpret CTA results with caution and, when necessary, proceed with confirmatory testing. Unfortunately, there remains a lack of high-quality prospective studies specifically addressing how CCS can be best incorporated into risk prediction models alongside CTA. Most current evidence is observational or retrospective, and there is a pressing need for large-scale, multicenter randomized trials to evaluate the clinical impact of CCS-guided decision-making [11,12]. Such trials should aim to determine whether CCS use leads to improved outcomes, reduced unnecessary testing, or more efficient allocation of healthcare resources [13]. The primary aim of our study, therefore, is to assess the correlation between CCS and the severity of CAD in patients undergoing coronary CTA. By analyzing the relationship between the quantitative calcium burden and the degree of luminal stenosis or plaque complexity, we hope to clarify the predictive value of CCS and determine its role in clinical management pathways. Specifically, we are interested in evaluating whether higher CCS values are consistently associated with more severe CAD, and whether CCS can serve as a gatekeeper for further invasive evaluation.

### 1.1. Study Population

Four hundred and five patients who underwent a coronary CT with CCS analysis were considered for this retrospective study. Patients with stent or bypass revascularization were excluded. Out of 405 patients, 217 were male. The mean and standard deviation age was 72 ± 11 years. Table 1 shows patients’ characteristics. The study was conducted in accordance with the Declaration of Helsinki and approved by the Local Ethics Committee Comitato Etico Milano area 2 (protocol code “FibroRetro”, number 1042_2022; approved on 22 November 2022).

### 1.2. Images Acquisition

Coronary CTA was performed using a dual-source (256-slice) CT scanner (SOMATOM Definition Flash, Siemens Healthcare, Forchheim, Germany). Before injecting the contrast medium, non-contrasted cardiac CT was performed in a longitudinal scan field from the tracheal carina down to the diaphragm. The corresponding images for calcium scoring were reconstructed with a slice width of 1.5 mm and slice interval of 1 mm, and the tube voltage was 120 kVp.

### 1.3. Images Analysis

**Figure 1:**
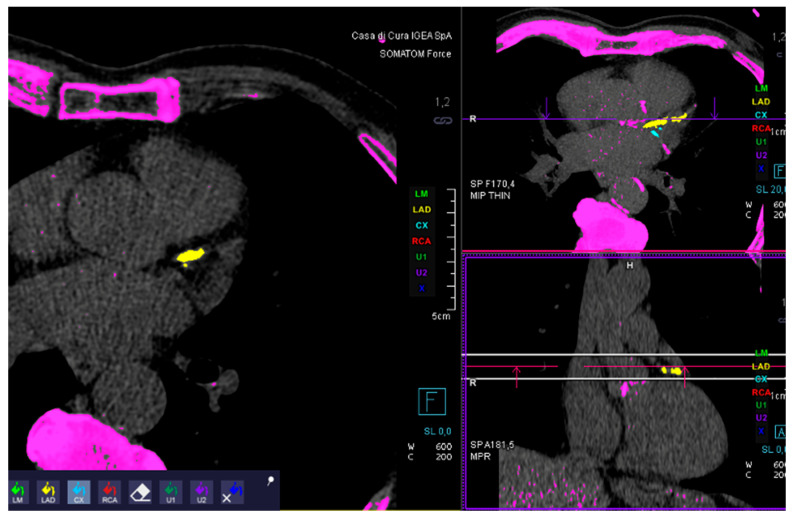
Example of coronary calcium score analysis.

The total calcium score was calculated using dedicated software (SyngoVia CT, version VB60A_HF08). The calcium score based on the Agatston method was defined as the presence of a lesion with an area greater than 1 mm^2^, and peak intensity greater than 130 Hounsfield Units, which was automatically identified and marked with color by the software (Figure 1). From the radiological report, the degree of coronary stenosis was retrieved. A score of 1 corresponds to the absence of stenosis, a score of 2 to mild stenosis (<50%), and a score of 3 to moderate/severe stenosis (>50%). The total coronary gravity score (CGS) is a study-specific definition (Table 2). CGS for each patient was calculated by summing the score of each coronary artery. The Spearman test was used for correlations.

### 1.4. Statistical Analysis

Data are expressed as mean ± standard deviation. Statistical analyses were performed using Python v. 3.7, and *p*-values ≤ 0.05 were considered to indicate statistical significance Each variable of interest was tested for normality using the Shapiro–Wilk test. Correlations between parameters were assessed using the Spearman test. A logistic regression model to identify the optimal cutoff point for CGS was used. The sample size was fixed, then a detectable effect size was calculated focusing on the most clinically relevant result as ROC analysis. To perform this analysis, Cohen’s d test was used.

## 2. Results

**Figure 2:**
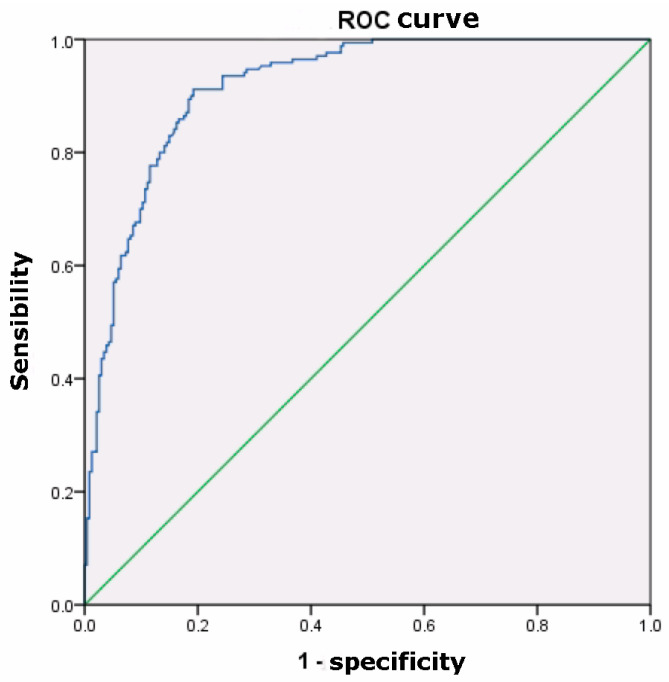
A ROC curve analysis is shown. AUC is 0.917 (*p* ≤ 0.001).

The overall calcium score was 393 ± 709 Agatston units. A CGS score of 4 was found in 69 patients, a score of 5 in 87 patients, a score of 6 in 78 patients, a score of 7 in 108 patients, a score of 8 in 44 patients, a score of 9 in 14 patients, and a score of 10 in 5 patients. A strong positive correlation was observed between CCS and CGS (r = 0.835, *p* < 0.001). Receiver operating characteristic (ROC) analysis yielded an AUC of 0.917 (IC = 0.878; 0.956) (*p* < 0.001). The optimal calcium cutoff score for discriminating CGS < 2 was 112, with a sensitivity of 90% (IC = 85; 95) and specificity of 81% (IC = 75; 88) (Figure 2). The positive predictive value (PPV) was 87.7% and the negative predictive value (NPV) was 84.4%. Cohen’s d test resulted in a value of 1.95, indicating a very large effect size. This suggests that the distributions of the test values for the positive and negative groups are almost completely separated, with minimal overlap between patients with significant coronary stenosis and patients without significant coronary stenosis.

## 3. Discussion

### 3.1. Background

The coronary calcium score, obtained through non-invasive imaging techniques such as CT, allows for the quantification of calcified plaque in the coronary arteries. Since coronary calcification is strongly associated with atherosclerotic disease, CCS provides a direct measure of subclinical atherosclerosis. Unlike traditional risk factors—which estimate the probability of coronary events indirectly—CCS offers a tangible representation of actual anatomical changes within the coronary vasculature. This capability makes CCS a particularly compelling addition to existing cardiovascular risk assessment models. In addition to its diagnostic and prognostic roles, CCS is also valuable for motivational purposes. Studies have shown that visualizing calcification in coronary arteries can have a profound psychological impact on patients, often leading to increased adherence to medication regimens, dietary improvements, and smoking cessation. This behavioral component further enhances the utility of CCS, making it not only a diagnostic tool but also a catalyst for lifestyle change and risk reduction. Nevertheless, while the utility of CCS is evident, there are important limitations and considerations that must be addressed. First, the technique primarily detects calcified plaque and may miss non-calcified or mixed plaques, which can also be prone to rupture and lead to acute coronary events. Therefore, a low CCS or a score of zero does not entirely rule out the presence of atherosclerosis, especially in younger patients or those with inflammatory conditions. In these cases, further imaging or functional testing may still be warranted. Second, radiation exposure, while relatively low with modern CT protocols, remains a consideration, particularly for repeated testing. As technology continues to advance, newer imaging techniques are likely to reduce radiation doses even further, making serial assessments more feasible in clinical settings. Moreover, disparities in access to imaging technology and the cost of coronary calcium scanning can pose barriers to widespread use. While CCS is generally cost-effective compared to invasive testing, the initial investment in equipment and training can be substantial, particularly in resource-limited settings. Health systems and policymakers should consider these factors when developing guidelines for CCS implementation. Looking ahead, future research should continue to explore the integration of CCS into routine cardiovascular risk assessment models, particularly in conjunction with emerging biomarkers, genetic risk scores, and machine learning algorithms. Combining anatomical data from CCS with functional, genetic, and biochemical markers could pave the way for a more comprehensive and individualized approach to cardiovascular prevention. Furthermore, longitudinal studies evaluating the impact of CCS-guided strategies on clinical outcomes, cost-effectiveness, and healthcare resource utilization are essential. Randomized controlled trials comparing CCS-based management with standard care would provide definitive evidence to support its broader application in everyday clinical practice. There is also growing interest in the use of CCS in special populations, including women, ethnic minorities, and individuals with conditions such as diabetes, chronic kidney disease, and HIV, where traditional risk models often underperform. Tailoring CCS interpretation to these subgroups could further enhance its predictive value and help close the gaps in cardiovascular care. Recent guideline updates and major reviews from the 2020s emphasize that CAC is a strong predictor of atherosclerotic cardiovascular disease events and can meaningfully reclassify individuals at intermediate risk; a CAC of ≥100 is commonly used as a threshold to consider initiation of statin therapy, whereas a CAC of 0 confers a low short- to medium-term event risk (the ‘power of zero’). Incorporating CAC staging alongside clinical risk scores (e.g., MESA-based tools/coronary age) has been recommended to personalize preventive strategies. These recommendations are supported by recent statements and reviews [14].

### 3.2. Data Discussion

The primary outcome of our study is the confirmation of the relationship between coronary calcium score and coronary gravity score with a significant positive correlation (r = 0.835 and *p* < 0.001). This result highlights the correlation between coronary artery calcium and the development of cardiovascular disease, pointing out that the CCS is a useful index of CAD. Coronary calcium scoring is a recognized and widely used method that allows quantitative assessment of calcifications as a predictor of cardiovascular events. CCS has been demonstrated to be a valuable prognostic tool for assessing the risk of developing cardiovascular diseases. Measuring calcium density can help in planning primary prevention and predicting the risk of mortality due to the evaluation of coronary involvement even in asymptomatic individuals. This study found out that the ideal cutoff point for the calcium score for discriminating coronary involvement (CGS 2) from a normal condition was 112. We defined a coronary gravity score of 2 as a mild risk of coronary artery stenosis (<50%). It is important to remember that CGS 1 and CGS 3 represent the absence of stenosis and severe risk of CAD, respectively. In these cases, coronary artery calcium scoring is known to be a less efficacious method of assessing the risk of major cardiac events. The absence of coronary artery calcification (calcium score of zero) does not exclude the presence of coronary artery disease, especially when there are significant risk factors [15]. Several studies regarding CCS as a valuable method for guiding the initiation or postponement of preventive therapies have shown that a coronary gravity score of 3 does not impact the treatment approach for this particular population [16].

Our study adds to this growing evidence base by confirming that higher calcium scores are not only associated with the presence of coronary artery disease but also correlate significantly with the extent and severity of disease, as represented by grading scales such as CGS. This correlation supports the use of CCS not only for risk prediction but also for disease monitoring and treatment planning. For instance, in patients with established risk factors or symptoms suggestive of angina, a high CCS may prompt further diagnostic evaluation using stress testing or coronary angiography, whereas a low or zero score may help to rule out significant obstructive disease. In terms of clinical decision-making, the integration of CCS into routine practice could lead to more personalized, evidence-based care. Clinicians could use CCS data to tailor treatment plans, avoiding both overtreatment in patients with low scores and undertreatment in patients with high scores who might otherwise be classified as low risk. In this way, CCS can support shared decision-making, where clinicians and patients collaborate to make informed choices based on a clearer understanding of individual risk.

The importance of finding a reliable cutoff point for CCS consists of providing the best balance between sensitivity and specificity [17]. As shown in Figure 2, this study yields acceptable values of sensitivity (90%) and specificity (81%). A similar study [17] identified an adequate cutoff point of 350, achieving a high negative predictive value (99%) while compromising on sensitivity (87%).

### 3.3. Limitations

This study presents certain limitation. First of all, this is a monocentric study. Secondly, we have no patient outcomes and no prognostic implications of CCS could be performed. Finally, our data is not be generalizable to other ethnic groups, and there may be potential racial or ethnic differences.

### 3.4. Clinical Perspectives

With the perspective of using CCS as a screening method that changes the clinical approach to the patient, it is important to introduce it into guidelines for risk assessment in asymptomatic patients. ROC curves for risk scores have been demonstrated to change when CCS is applied within the MESA (Multi-Ethnic Study of Atherosclerosis) cohort [18,19].

The coronary calcium score has emerged as a noninvasive and consistent screening method to detect the presence and extension of coronary artery calcification that correlates with CTA. It is associated with reliable cardiovascular risk stratification, dose reduction, and cost effectiveness in asymptomatic patients [20].

## 4. Conclusions

In conclusion, this study reinforces the strong relationship between CCS and both the presence and extent of coronary artery disease. The significant positive correlation observed between CCS and CAD highlights the potential of coronary calcium scoring as a valuable and accessible diagnostic tool for assessing the severity of coronary involvement. Finally, we suggest an Agatston score of 112 as an optimal calcium score cutoff for discriminating mild CAD.

## Figures and Tables

**Table 1:** Patients’ characteristics.

Male	Female	Age	Smoke	Hypertension	Hypercolesterolemia
217	188	72 ± 11	76%	65%	59%

**Table 2:** Coronary gravity score (CGS).

CGS	1	2	3
	No stenosis	Mild stenosis	Moderate/severe stenosis

## Data Availability

The data presented in this study are available upon reasonable request from the corresponding author. The data are not publicly available due to privacy concerns.

## Abbreviations

CTA	Computed Tomography Angiography
CCS	Coronary Calcium Score
CGS	Coronary Gravity Score
CT	Computed Tomography
